# Supplementary material for: A calcium/cAMP signaling loop at the ORAI1 mouth drives channel inactivation to shape NFAT induction
Source: Nat Commun. 2019 Apr 29;10:1971. doi: 10.1038/s41467-019-09593-0 (PMC6488650; doi:10.1038/s41467-019-09593-0)
Supplement: Supplementary file 1 — Supplementary Information [file 41467_2019_9593_MOESM1_ESM.pdf]

Supplementary Information

A Calcium/cAMP Signaling Loop at the ORAI1 Mouth Drives Channel Inactivation to Shape NFAT Induction

Zhang et al.

Supplementary Table 1. Key resources and reagents used in study

Reagent or Resource	Source	Identifier
Antibodies		
Rabbit polyclonal anti-ORAI1	Sigma-Aldrich, now MilliporeSigma	Cat#O8264
Mouse monoclonal anti-GAPDH	MilliporeSigma	Cat#MAB374
Rabbit polyclonal anti-ADCY8	Labome	Cat#55065-1-AP
Mouse monoclonal anti-GFP	Santa Cruz Biotechnology	Cat#sc-9996
Rabbit polyclonal anti-Phosphoserine	MilliporeSigma	Cat#AB1603
Mouse monoclonal anti-PPP3R1	Santa Cruz Biotechnology	Cat#sc-130393
Mouse monoclonal anti-HSC70	Santa Cruz Biotechnology	Cat#sc-24
Chemicals and Peptides		
Thapsigargin	Calbiochem	Cat#586005
[Ethylenebis-(oxyethylenenitrilo)]-Tetraacetic Acid (EGTA)	Thermo Fisher Scientific	Cat#O2783-100
Carbachol	Sigma-Aldrich	Cat#212385-100MG-M
Forskolin	Calbiochem	Cat#344273-10MG
Adenosine 3′,5′-cyclic monophosphate (cAMP)	Sigma-Aldrich	Cat#A9501-1G
H-89	abcam	Cat#ab143787
FK506 (Tacrolimus)	abcam	Cat#ab120223
Gö6983	abcam	Cat#ab144414
KT5720	MilliporeSigma	Cat#420320-100UG
KT5823	MilliporeSigma	Cat#420321-100UG
St-Ht31	Tocris Bioscience	Cat#6286
St-Ht31 P	Tocris Bioscience	Cat#6287
Cyclosporin A	Cayman Chemical	Cat#12088
8-pCPT-2-O-Me-cAMP-AM	Tocris Bioscience	Cat#4853
Phosphatidylinositol 4,5-bisphosphate diC8 (PI(4,5)P_2 diC8)	Echelon Biosciences	Cat#P-4508
Critical Commercial Assays		
Dynabeads co-immunoprecipitation kit	Invitrogen	Cat#14321D
Guide-it Mutation Detection Kit	Clontech Laboratories	Cat#631443
Cell line Nucleofector Kit	Lonza	Cat#VCA-1003

Experimental Models: Cell Lines		
HEK293 cells	ATCC	CRL-1573
ORAI1 knock-out HEK293 cells	Trebak Lab; This paper	N/A
STIM1 knock-out HEK293 cells	Trebak Lab; This paper	N/A
STIM2 knock-out HEK293 cells	Trebak Lab; This paper	N/A
STIM1/2 double knock-out HEK293 cells	Trebak Lab; This paper	N/A
Oligonucleotides		
AllStars Neg. Control siRNA	Qiagen	Cat#1027281
Human ADCY8 siRNA; for detailed information see Supplementary Table 3	Dharmacon (ON-TARGETplus)	Cat#J-006637-11-0005; LU-006637-01- 0002
Human PPP3R1 siRNA; for detailed information see Supplementary Table 3	Integrated DNA Technologies	N/A
Software and Algorithms		
Origin 9.0	OriginLab	https://www.originlab.com/
Clampfit 10.3	Molecular Devices, LLC.	https://www.moleculardevices.com/
ImageJ	Schneider et al., 2012	https://imagej.nih.gov/ij/
InCytim2 V5.35	INTRACELLULAR IMAGING	http://www.intracellular.com/products2.html
LAS X Life Science Microscope Software	Leica	https://www.leica-microsystems.com/
SlideBook 6.0	Intelligent Imaging Innovations, Inc.	https://www.intelligent-imaging.com/slidebook
MAXCHELATOR	Chris Patton: cpatton@stanford.edu	http://maxchelator.stanford.edu/
Recombinant DNA		
CMV-WT-ORAI1-EGFP	Putney Lab	N/A
CMV-ORAI1-EGFP	Putney Lab	N/A
CMV-ORAI1β-EGFP	Putney Lab	N/A
CMV-R31,32,33A-ORAI1-EGFP	Putney Lab	N/A
CMV-Y52A,W55A-ORAI1-EGFP	Putney Lab	N/A
TK-WT-ORAI1-YFP	Putney Lab	N/A
TK-ORAI1-YFP	Putney Lab	N/A
TK-ORAI1β-YFP	Putney Lab	N/A
EYFP-STIM1	Meyer Lab	N/A
STIM1-pVenus	Shuttleworth Lab	N/A
T389A-STIM1-pVenus	Shuttleworth Lab	N/A

T389E-STIM1-pVenus	Shuttleworth Lab	N/A
CMV-S27A,S30A-ORAI1	Trebak Lab; This paper	N/A
Non-tagged AC8	Trebak Lab; This paper	N/A
CFP-AC8	Cooper Lab	N/A
CMV-YFP-AC8	Cooper Lab	N/A
GCaMP2-AC8M1	Cooper Lab	N/A
CMV-ORAI1-CFP	Trebak Lab; This paper	N/A
CMV-ORAI1β -CFP	Trebak Lab; This paper	N/A
CMV-EYFP-ORAI1	Trebak Lab; This paper	N/A
CMV-EYFP-ORAI1β	Trebak Lab; This paper	N/A
CMV-AKAP79-GFP	Origene	RG221314
CMV-(Δ1-107)AKAP79-GFP	Trebak Lab; This paper	N/A
CMV-S34A-ORAI1	Trebak Lab; This paper	N/A
CMV-S34D-ORAI1	Trebak Lab; This paper	N/A
CMV-S34E-ORAI1	Trebak Lab; This paper	N/A
CMV-S34R-ORAI1	Trebak Lab; This paper	N/A
TK-S34A-ORAI1	Trebak Lab; This paper	N/A
TK-S34D-ORAI1	Trebak Lab; This paper	N/A
TK-S34E-ORAI1	Trebak Lab; This paper	N/A
TK-S34R-ORAI1	Trebak Lab; This paper	N/A
TK-ORAI1-EGFP	Trebak Lab; This paper	N/A
TK-ORAI1β-EGFP	Trebak Lab; This paper	N/A
HA-NFAT1(4-460)-GFP	Addgene	Cat#11107
HA-NFAT4(3-407)-GFP	Addgene	Cat#21664
LentiCRISPR v2	Feng Zhang, Addgene	Cat#52961

Supplementary Table 2. Primers for real-time PCR and primers used for cloning in this study

Primers	Forward	Reverse
GAPDH (real-time PCR)	5’-CCCTTCATTGACCTCAACTACA-3’	5’-ATGACAAGCTTCCCGTTCTC-3’
PPP3R1 (real-time PCR)	5’-TCCAGCAATGCTCTCTGTGT-3’	5’-CCTTCCCTTTCTCCACCACA-3’
PPP3R2 (real-time PCR)	5’-ATGGGAAACGAGGCCAGTTA-3’	5’-TGTCGAAGACGTCGATCACT-3’

STIM1 KO-CRISPR/Cas9	CAC CGT GAT GAG CTT ATC CTC ACC A	AAA CTG GTG AGG ATA AGC TCA TCA C
STIM2 KO- CRISPR/Cas9	CAC CGA GAT GGT GGA ATT GAA GTA G	AAA CCT ACT TCA ATT CCA CCA TCT C
ORAI1 KO- CRISPR/Cas9	CAC CGG TTG CTC ACC GCC TCG ATG T	AAA CAC ATC GAG GCG GTG AGC AAC C
Long ORAI1 (from WT-ORAI1-CFP)	CGG CGG CCA CCA TGC ATC CGG AGC CCG CC	GCA TGG TGG CCG CCG CCG AGG CGC GGG C
ORAI1β (from WT-ORAI1-CFP)	GGC GTG CTC CGC GCA TCC GGA GCC CGC CC	GCT CCG GAT GCG CGG AGC ACG CCG CCG AG
CMV-S27A,S30A-ORAI1 mutation	GCG CTC GCC GGG CTC GCC GCC GCA GCG GGG A	GCG AGC CCG GCG AGC GCC GCT GGT GGT GCT GC
Non-tagged AC8 (Remove YFP tag from YFP-AC8)	CCA CCA TGG ACG GAG GCG GGG AAC TCT	CTC CGT CCA TGG TGG GCT AGC GGA TCT
eGFP to CFP pair1 for xFP	AAG GGC GAG GAG CTG TTC A	TCG TCC ATG CCG AGA GTG AT
eGFP to CFP pair2 for vector	AGC TCC TCG CCC TTG CTC A	TCT CGG CAT GGA CGA GCT GT
CMV-(Δ1-107)AKAP79-GFP truncation	TCG CCA TGA TAA ATG CTG AGG ATG CTG ATC	CAT TTA TCA TGG CGA TCG CGG CGG CA
CMV-S34A-ORAI1 mutation	CGC CGC GCA GGG GAC GGG GAG CCC	GTC CCC TGC GCG GCG GCG GCT CCG
CMV-S34D-ORAI1 mutation	CGC CGC GAT GGG GAC GGG GAG CCC	GTC CCC ATC GCG GCG GCG GCT CCG
CMV-S34E-ORAI1 mutation	CGC CGC GAA GGG GAC GGG GAG CCC	GTC CCC TTC GCG GCG GCG GCT CCG
CMV-S34R-ORAI1 mutation	CGC CGC AGA GGG GAC GGG GAG CCC	GTC CCC TCT GCG GCG GCG GCT CCG
For TK-S34A/D/E/R-ORAI1s (Change from CMV promoter to TK promoter using CMV-S34A/D/E/R-ORAI1s) pair1	CGG GGT CTA AAT GAG TCT TCG GAC CTC GC	CAT GGT GGG CTA GCC TAT AGT GAG TCG TAT T
For TK-S34A/D/E/R-ORAI1s (Change from CMV promoter to TK promoter using CMV-S34A/D/E/R-ORAI1s) pair2 for vector	CGC AAA TGG GCG GTA GGC GTG	CTC ATT TAG ACC CCG TAA TTG ATT ACT A

Supplementary Table 3. List of siRNA sequences

SiRNAs	Sequence	Source
Control siRNA	Commercial product (N/A)	Integrated DNA Technologies
siPPP3R1#1	GCAAGUUAUCCUUUGGAA	Integrated DNA Technologies
siPPP3R1#2	CCUUUAGUACAGCGAGUA	Integrated DNA Technologies
siPPP3R2	GGUUUAAGAAGUUGGAC	Integrated DNA Technologies
siAC8#1	Commercial product (N/A)	Dharmacon (ON-TARGETplus)
siAC8#2	Commercial product (N/A)	Dharmacon (ON-TARGETplus)

Supplementary Table 4. Statistical analysis of patch clamp data from this study

Figure	Experiment	Stimulus	Value@-120 (Mean±SEM)	Value@-100 (Mean±SEM)	Value@-80 (Mean±SEM)	Value@-60 (Mean±SEM)
1d	ORAI1	EGTA	0.49412±0.00612	0.54987±0.0081	0.62994±0.0095	0.70687±0.01119
	ORAI1β	EGTA	0.74078±0.01279	0.78022±0.01177	0.81248±0.01101	0.83604±0.01107
1g	ORAI1	BAPTA	0.73783±0.0082	0.76367±0.00632	0.79817±0.00636	0.82317±0.00609
	ORAI1β	BAPTA	0.75333±0.01387	0.77867±0.01416	0.80133±0.01547	0.81667±0.00996
1j	ORAI1	Ba^{2+}	0.91017±0.01384	0.98367±0.00685	1.00283±0.01186	0.95917±0.03025
	ORAI1β	Ba^{2+}	0.91575±0.02295	0.96337±0.02637	0.98125±0.02521	0.94175±0.02083
1n	ORAI1	None	0.49375±0.00781	0.5471±0.00982	0.61925±0.01305	0.68435±0.01401
	R31,32,33A-ORAI1	None	0.53364±0.01277	0.594±0.01197	0.66855±0.0148	0.73773±0.01441
1o	ORAI1	None	0.51656±0.00774	0.57911±0.00926	0.66194±0.01169	0.74728±0.01383
	Y52A,W55A-ORAI1	None	0.64575±0.01109	0.70117±0.01253	0.75446±0.01349	0.805±0.01311
2c	ORAI1, siCon	None	0.494±0.01206	0.54433±0.01039	0.63±0.0127	0.72022±0.01054
	ORAI1, SiAC8#2	None	0.55825±0.00902	0.6145±0.00982	0.7075±0.01107	0.79112±0.01424
2i	ORAI1, + empty vector	None	0.49412±0.00612	0.54987±0.0081	0.62994±0.0095	0.70687±0.01119
	ORAI1, + AC8	None	0.43879±0.00965	0.491±0.00994	0.55289±0.0098	0.61837±0.00947
	ORAI1, +AC8M1	None	0.50425±0.00646	0.55812±0.00716	0.63694±0.00836	0.70862±0.01067
3c	ORAI1	Vehicle_bath	0.48715±0.00704	0.54037±0.00812	0.61733±0.01043	0.68678±0.01183
	ORAI1	10 µM Forskolin	0.44408±0.00906	0.5025±0.00704	0.5615±0.00852	0.63417±0.01191
3f	ORAI1	Vehicle_ pipette	0.49375±0.00781	0.5471±0.00982	0.61925±0.01305	0.68435±0.01401
	ORAI1	100 µM cAMP	0.44583±0.01053	0.49317±0.01302	0.558±0.015	0.62725±0.01635
3i	ORAI1	Vehicle Con	0.50206±0.00849	0.56094±0.00958	0.64087±0.01146	0.72087±0.01196
	ORAI1	10 µM H89	0.56233±0.01544	0.62508±0.01797	0.70825±0.01431	0.80075±0.01334
3l	ORAI1	100 µM cAMP	0.45008±0.01103	0.50046±0.01259	0.56477±0.01602	0.64238±0.02005

	ORAI1	100 µM cAMP + 10 µM H89	0.5702±0.00885	0.63593±0.00909	0.73±0.0105	0.81853±0.0142
3p	ORAI1, + empty vector	10 µM Forskolin	0.4465±0.00711	0.511±0.00532	0.60833±0.01821	0.7275±0.02685
	ORAI1, + AKAP79	10 µM Forskolin	0.3967±0.00852	0.4455±0.01076	0.5274±0.01397	0.606±0.01909
	ORAI1, + (Δ1-107)AKAP79	10 µM Forskolin	0.4664±0.01217	0.5187±0.01276	0.6142±0.01211	0.7109±0.01443
4c	ORAI1	10 µM st-Ht31 P	0.48625±0.0071	0.53787±0.00955	0.61625±0.00772	0.69825±0.00559
	ORAI1	10 µM st-Ht31	0.55292±0.00843	0.59715±0.0078	0.66023±0.00867	0.73962±0.00689
4i	ORAI1	None	0.49375±0.00781	0.5471±0.00982	0.61925±0.01305	0.68435±0.01401
	S34A-ORAI1	None	0.55894±0.01496	0.60419±0.01467	0.67806±0.01229	0.74969±0.01396
	S34R-ORAI1	None	0.61555±0.01901	0.66527±0.02278	0.72036±0.02099	0.78118±0.02147
	S34D-ORAI1	None	0.46583±0.01294	0.5215±0.01194	0.59833±0.01149	0.68017±0.0093
	S34E-ORAI1	None	0.488±0.01363	0.54671±0.01739	0.60414±0.01566	0.664±0.01744
4o	ORAI1	Vehicle con	0.50875±0.00542	0.56125±0.00867	0.6315±0.00658	0.7115±0.00656
	ORAI1	1 µM FK506	0.42225±0.00857	0.46263±0.01136	0.52163±0.00551	0.60113±0.00965
	ORAI1	100 nM CsA	0.4385±0.01497	0.47483±0.01913	0.52283±0.01857	0.59±0.01257
4s	ORAI1, siCon	None	0.49387±0.01248	0.5405±0.01207	0.61037±0.00969	0.6905±0.0098
	ORAI1, siPPP3R1#1	None	0.4166±0.00997	0.4646±0.00884	0.5274±0.01076	0.6174±0.01251
	ORAI1, siPPP3R1#2	None	0.40913±0.0097	0.46±0.01025	0.52338±0.01212	0.61587±0.00535
S1g	ORAI1	None	5.07212±0.12826	5.00105±0.32764	4.43937± 0.42781	---
	ORAI1β	None	1.76±0.169	1.71±0.317	1.61±0.453	---
S1h	ORAI1	None	35.83523±0.96816	35.50037±1.57039	30.34671±1.8287	---
	ORAI1β	None	13.31±1.21	9.19±1.67	3.73±0.789	---
S1k	ORAI1	None	0.49375±0.00781	0.5471±0.00982	0.61925±0.01305	0.68435±0.01401
	S27A,S30A-ORAI1	None	0.50227±0.00991	0.55936±0.01107	0.62891±0.01359	0.70491±0.01425
S1n	ORAI1	Vehicle con	0.4971±0.01263	0.5537±0.01413	0.6355±0.01723	0.7168±0.01856
	ORAI1	10 µM Go6983	0.52233±0.012	0.57273±0.01202	0.65173±0.01218	0.72753±0.01163
S2c	ORAI1	Vehicle con	0.49375±0.00781	0.5471±0.00982	0.61925±0.01305	0.68435±0.01401

	ORAI1	30 µM PIP_2	0.4953±0.00364	0.5486±0.00831	0.6107±0.01157	0.679±0.0191
S2f	siNT, R31,32,33A-ORAI1	None	0.5355±0.01558	0.5935±0.01055	0.68638±0.01165	0.7665±0.01522
	siAC8#2, R31,32,33A-ORAI1	None	0.53537±0.00753	0.58988±0.01161	0.67388±0.01432	0.76875±0.01785
S2i	siCon, ORAI1β	None	0.6777±0.00862	0.7268±0.00682	0.7852±0.00553	0.8431±0.00605
	siAC8#1, ORAI1β	None	0.68182±0.01412	0.73764±0.01655	0.80909±0.0161	0.85536±0.01172
S2l	siCon, ORAI1	None	0.494±0.01206	0.54433±0.01039	0.63±0.0127	0.72022±0.01054
	siAC8#1, ORAI1	None	0.54358±0.00755	0.60058±0.00813	0.69153±0.00861	0.78268±0.00951
S3c	ORAI1β	Vehicle con	0.68975±0.01679	0.73694±0.01782	0.79281±0.01776	0.83044±0.0155
	ORAI1β	10 µM Forskolin	0.688±0.02874	0.74343±0.02365	0.79143±0.02241	0.82229±0.01259
S3f	ORAI1β	Vehicle con	0.68975±0.01679	0.73694±0.01782	0.79281±0.01776	0.83044±0.0155
	ORAI1β	100 µM cAMP	0.67465±0.01416	0.73022±0.01619	0.77765±0.01601	0.81157±0.01638
S4c	ORAI1	Vehicle con	0.4989±0.00698	0.55715±0.00788	0.6417±0.00938	0.72495±0.01029
	ORAI1	10 µM 8-pCPT-AM	0.4882±0.00551	0.5411±0.00617	0.6139±0.00517	0.7037±0.00445
S4f	ORAI1	100 µM cAMP	0.45008±0.01103	0.50046±0.01259	0.56477±0.01602	0.64238±0.02005
	ORAI1	100 µM cAMP + 5 µM KT5720	0.51425±0.01185	0.57075±0.0092	0.66025±0.00912	0.7385±0.01471
S4i	ORAI1	Vehicle con	0.5006±0.01863	0.557±0.01062	0.6568±0.01363	0.7278±0.02933
	ORAI1	5 µM KT5823	0.509±0.02279	0.5605±0.02671	0.64233±0.02879	0.71617±0.03567
S5d	ORAI1, + empty vector	None	0.47133±0.01235	0.53367±0.01736	0.63733±0.02413	0.733±0.00814
	ORAI1, + AKAP79	None	0.4859±0.0098	0.5422±0.01022	0.6332±0.01378	0.7292±0.01492
	ORAI1, + (Δ1-107)AKAP79	None	0.49333±0.01879	0.54356±0.01879	0.62789±0.02388	0.72156±0.02637
S6g	ORAI1 + WT-STIM1	None	0.505±0.01332	0.56175±0.01371	0.63575±0.01422	0.723±0.01774
	ORAI1 +	None	0.50415±0.00529	0.55892±0.00632	0.63677±0.01035	0.69831±0.01269

	T389A-STIM1					
	ORAI1 + T389E-STIM1	None	0.51167±0.01844	0.56433±0.01795	0.63667±0.01859	0.702±0.00757
S6k	ORAI1	100 µM cAMP	0.4546±0.00452	0.505±0.00625	0.5808±0.00326	0.6424±0.008
	S34A-ORAI1	Vehicle con	0.5785±0.01687	0.61625±0.01215	0.68725±0.01388	0.76125±0.01121
	S34A-ORAI1	100 µM cAMP	0.59389±0.01213	0.64767±0.01446	0.71256±0.00965	0.78378±0.01334
S7d	ORAI1	Ba^{2+}	0.91017±0.01384	0.98367±0.00685	1.00283±0.01186	0.95917±0.03025
	S34A-ORAI1	Ba^{2+}	0.90167±0.00719	0.97083±0.01274	1.0025±0.02715	0.97283±0.03748
	S34E-ORAI1	Ba^{2+}	0.865±0.00558	0.97767±0.00543	1.03983±0.01381	1.0325±0.02276
S8c	S34A-ORAI1	10 mM EGTA	0.55894±0.01496	0.60419±0.01467	0.67806±0.01229	0.74969±0.01396
	S34A-ORAI1	20 mM BAPTA	0.72913±0.01431	0.75162±0.01341	0.77375±0.01479	0.7965±0.01387
S9d	ORAI1	10 mM EGTA	0.49412±0.00612	0.54987±0.0081	0.62994±0.0095	0.70687±0.01119
	ORAI1β	10 mM EGTA	0.74078±0.01279	0.78022±0.01177	0.81248±0.01101	0.83604±0.01107
	S34A-ORAI1	10 mM EGTA	0.55894±0.01496	0.60419±0.01467	0.67806±0.01229	0.74969±0.01396
S9g	ORAI1	1 mM EGTA	0.4116±0.01109	0.4413±0.01088	0.4899±0.01344	0.549±0.01597
	ORAI1β	1 mM EGTA	0.49391±0.02851	0.51182±0.02632	0.54073±0.02582	0.58609±0.02577
	S34A-ORAI1	1 mM EGTA	0.45013±0.02032	0.47587±0.02446	0.51712±0.02204	0.57313±0.02583

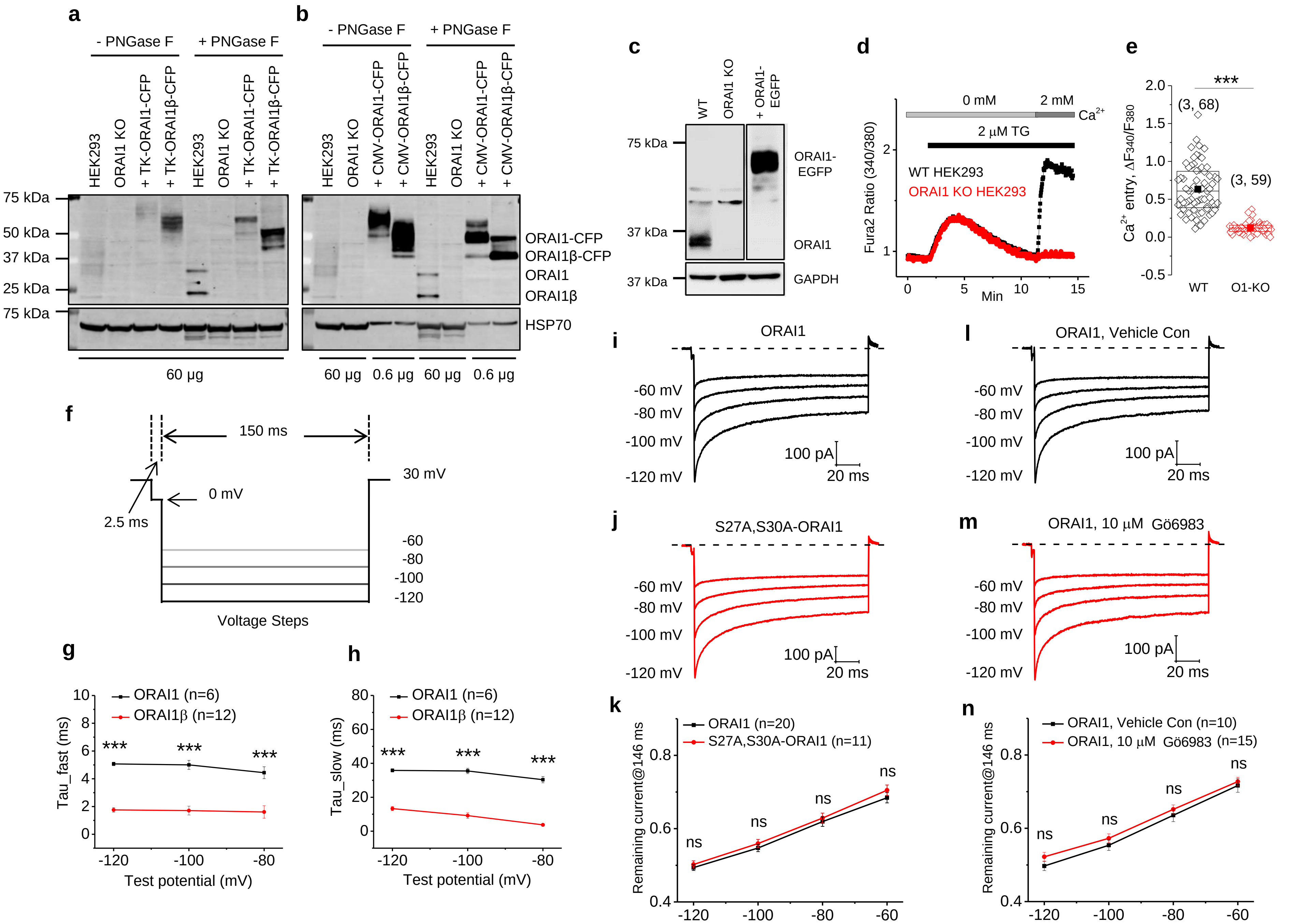

Supplemental Figure 1

Supplementary Figure 1. Protein kinase C (PKC) activity does not mediate ORAI1 CDI.

(a-c) Western blots with ORAI1 specific antibody on deglycosylated (+PNGase F) and non-treated proteins extracts from WT HEK293 cells, ORAI1-KO cells and ORAI1-KO cells expressing either ORAI1-CFP or ORAI1β-CFP driven by either thymidine kinase (TK) promoter (a) or cytomegalovirus (CMV) promoter (b). Note for CMV-driven vectors only 0.6 µg of proteins were loaded per well. (c) Anti-ORAI1 western blot, which was overexposed to document ORAI1 deletion. Protein extracts were from parental WT HEK293 cells, ORAI1-KO cells and ORAI1-KO cells expressing ORAI1-CFP.

(d, e) (d) Representative traces of Fura2 measurements of SOCE stimulated with 2µM thapsigargin (TG), and (e) Averaged Ca^{2+} entry calculated as increase in Fura2 ratio over basal values upon re-introduction of 2 mM Ca^{2+} to the bath solution (Δ340/380) in HEK293 WT and ORAI1-KO. For each group the number of cells is n = 68 and 59, respectively from a total of 3 independent experiments. While Ca^{2+} entry is abrogated in ORAI1-KO cells, Ca^{2+} release from internal stores is comparable to WT HEK293 cells. Boxplots show the mean, median, and the 75th to 25th percentiles.

(f) Voltage step protocol used to study ORAI1 CDI. Hyperpolarizing voltage steps lasting 150 ms were applied every 2 s from a holding potential of +30 mV. An initial 2.5 ms step from the holding potential to 0mV was applied to gauge and subtract potential contamination of peak current by cell capacitance.

(g, h) Current data were fitted to a biexponential function: $I = I_o + A_1e^{-t/\tau1} + A_2e^{-t/\tau2}$, where τ1 and τ2 are fast and slow time constants of inactivation. These (g) fast and (h) slow time constants were plotted against test potential. Each point represents mean ± SEM for *n* = 6-12 cells.

(i-k) Representative currents from ORAI-KO cells co-expressing eYFP-STIM1 with either (i) WT ORAI1-CFP, or (j) S27A, S30A ORAI1-CFP mutant, in which two Serine PKC phosphorylation sites are mutated to Alanine. (k) The extent of CDI is represented as current remaining at 146 ms. Each data point represents mean ± SEM for *n* = 11-20 cells.

(l-n) Representative currents from ORAI-KO cells co-expressing eYFP-STIM1 with ORAI1-CFP, and either treated in the bath with (l) control vehicle or (m) the PKC inhibitor Gö6983 (10 µM). (n) The extent of CDI is represented as current remaining at 146ms. Each point represents mean ± SEM for *n* = 10-15 cells.

***p<0.001; ns, not significant; two-tailed Student’s t test was used for (e, g, h, k, n).

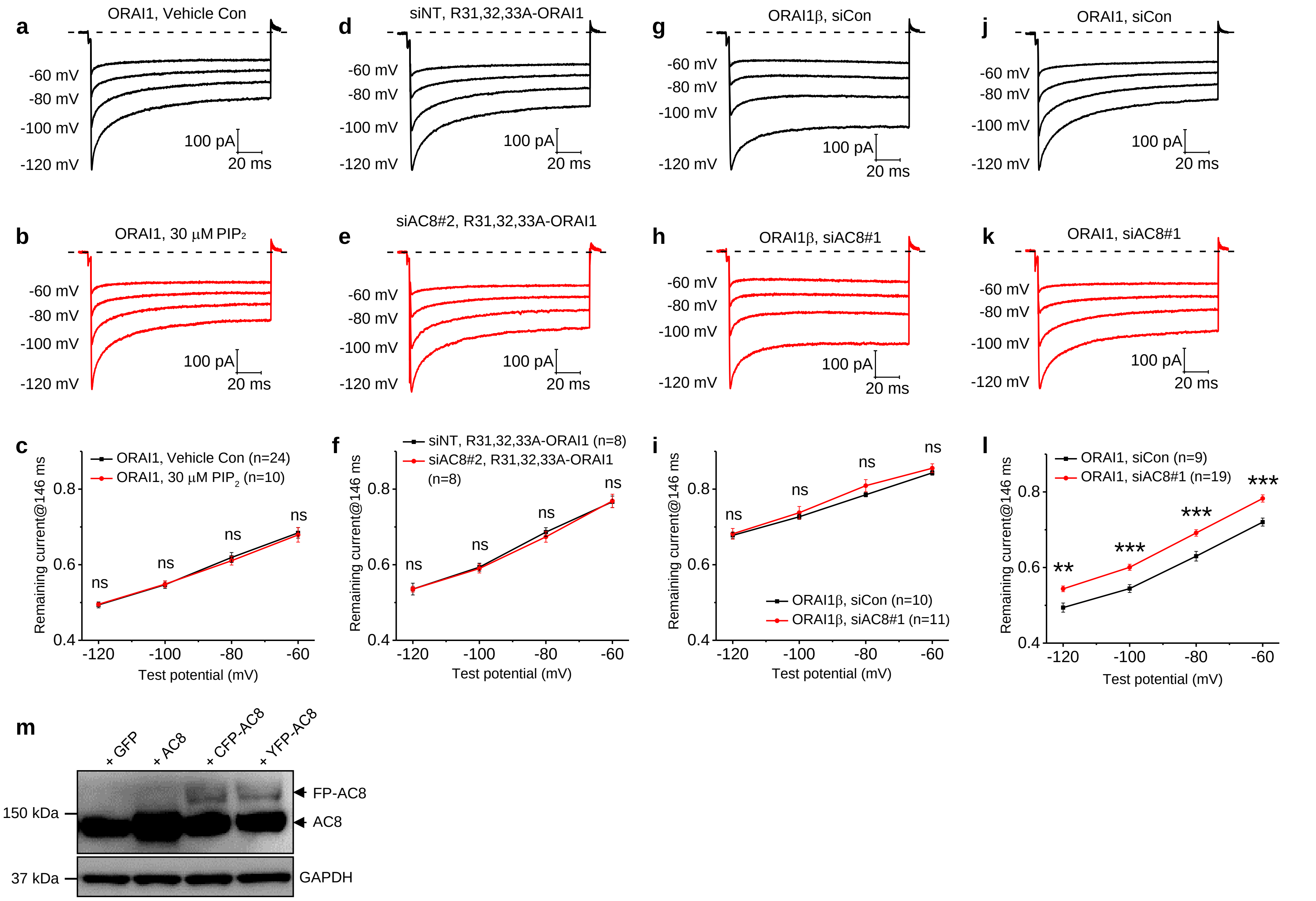

Supplemental Figure 2

Supplementary Figure 2. AC8 and cAMP do not mediate ORAI1β CDI.

(a-c) Representative currents from ORAI-KO cells co-expressing eYFP-STIM1 with ORAI1-CFP, and either dialyzed through the patch pipette with (a) control vehicle or (b) diC8-PIP_2 (30 µM). (c) The extent of CDI is represented as current remaining at the end of the pulse. Each data point represents mean ± SEM for n = 10-24 cells.

(d-f) Representative currents from ORAI-KO cells co-expressing eYFP-STIM1 with R31-33A ORAI1-CFP mutant deficient in AC8 binding, and either transfected with (d) control non-targeting siRNA or (e) siRNA against AC8. (f) The extent of CDI is represented as current remaining at 146 ms. Each data point represents mean ± SEM for n = 8 cells.

(g-i) Representative currents from ORAI-KO cells co-expressing eYFP-STIM1 with ORAI1β-CFP, and either transfected with (g) control non-targeting siRNA or (h) siRNA against AC8. (i) The extent of CDI is represented as current remaining at 146 ms. Each data point represents mean ± SEM for n = 10-11 cells.

(j-l) Representative currents (from experiments performed side by side with those in (g-i)) from ORAI-KO cells co-expressing eYFP-STIM1 with ORAI1-CFP, and either transfected with (j) control non-targeting siRNA or (k) siRNA against AC8. (l) The extent of CDI is represented as current remaining at 146 ms. Each data point represents mean ± SEM for n = 9-19 cells.

(m) Western blot with Anti-AC8 antibody in HEK293 cells expressing either GFP, untagged AC8, CFP-AC8 and eYFP-AC8.

**p<0.01; ***p<0.001; ns, not significant; two-tailed Student’s t test was used for (c, f, i, l).

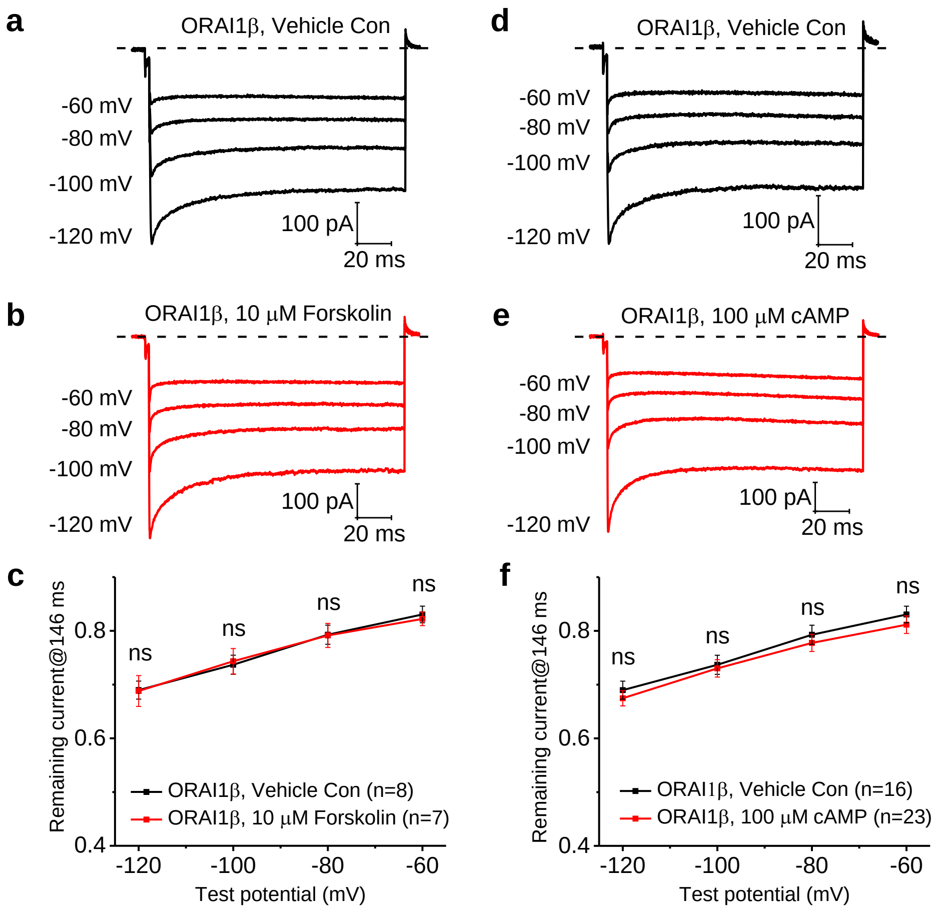

Supplemental Figure 3

Supplementary Figure 3. ORAI1β CDI is not affected by addition of forskolin (in bath) or cAMP (in pipette).

(a-c) Representative currents from ORAI-KO cells co-expressing eYFP-STIM1 with ORAI1β-CFP, and either treated in the bath with (a) vehicle control or (b) Forskolin (10 µM). (c) The extent of CDI is represented as current remaining at 146 ms. Each point represents mean ± SEM for *n* = 7-8 cells. ns, not significant; two-tailed Student's t test was used.

(d-f) Representative currents from ORAI-KO cells co-expressing eYFP-STIM1 with ORAI1β-CFP, and either dialyzed through the patch pipette with (d) vehicle control or (e) cAMP (100 µM). (f) The extent of CDI is represented as current remaining at 146 ms. Each point represents mean ± SEM for *n* = 16-23 cells. ns, not significant; two-tailed Student's t test was used.

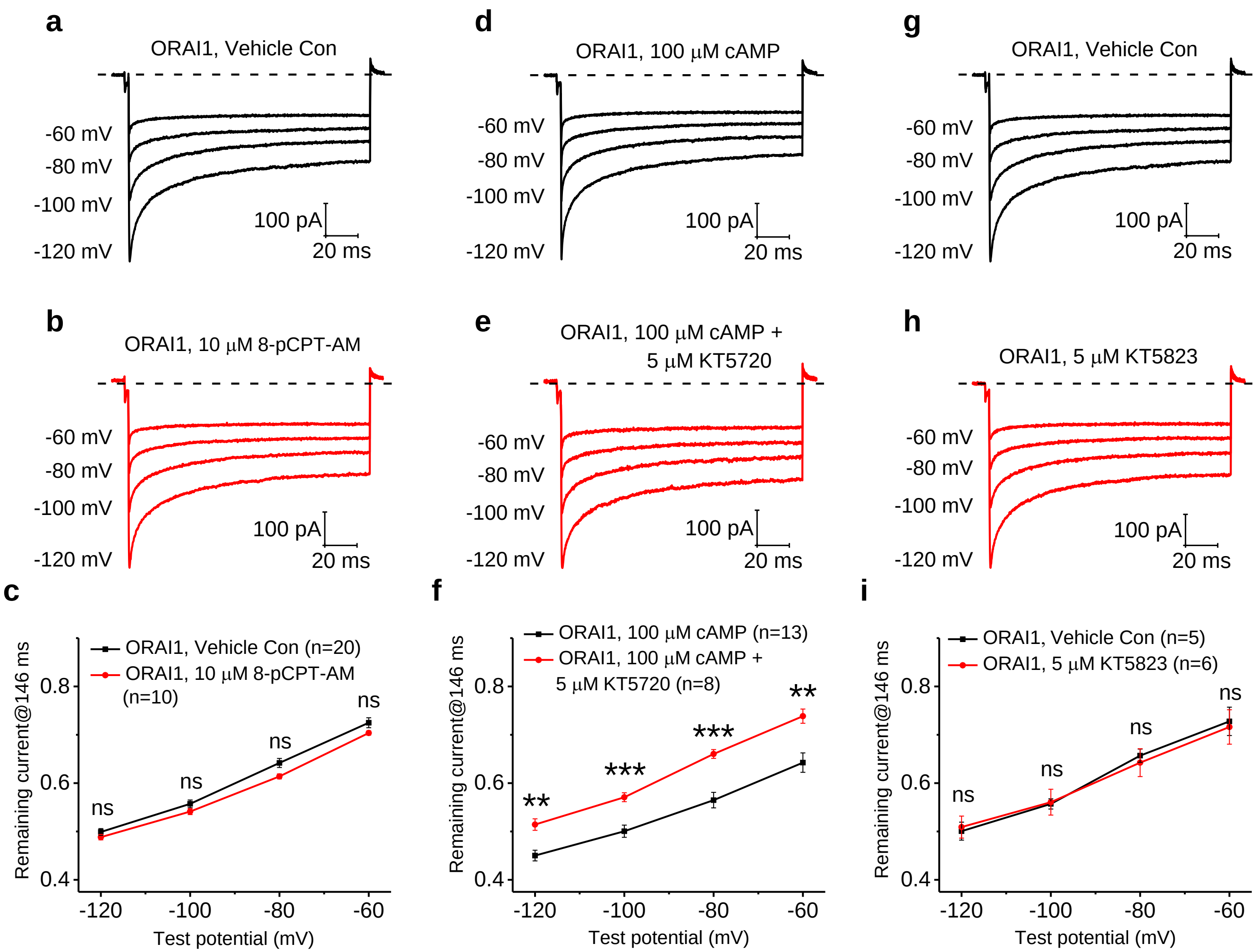

Supplemental Figure 4

Supplementary Figure 4. EPAC proteins and protein kinase G (PKG) do not mediate ORAI1 CDI.

(a-c) Representative currents from ORAI-KO cells co-expressing eYFP-STIM1 with ORAI1-CFP, and either treated in the bath solution with (a) vehicle control or (b) the membrane-permeant EPAC activator (8-pCPT-AM; 10µM). (c) The extent of CDI is represented as current remaining at 146 ms. Each point represents mean ± SEM for *n* = 10-20 cells. ns, not significant; two-tailed Student's t test was used.

(d-f) Representative currents from ORAI-KO cells co-expressing eYFP-STIM1 with ORAI1-CFP dialyzed with 100µM cAMP through the patch pipette, and treated in the bath with either (d) vehicle control or (e) the PKA inhibitor KT5720 (5 µM). (f) The extent of CDI is represented as current remaining at 146 ms. Each point represents mean ± SEM for *n* = 8-13 cells. **p<0.01; ***p<0.001; two-tailed Student's t test was used.

(g-i) Representative currents from ORAI-KO cells co-expressing eYFP-STIM1 with ORAI1-CFP, and either treated in the bath with either (g) vehicle control or (h) the PKG inhibitor KT5823 (5 µM). (i) The extent of CDI is represented as current remaining at 146 ms. Each point represents mean ± SEM for *n* =5-6 cells. ns, not significant; two-tailed Student's t test was used.

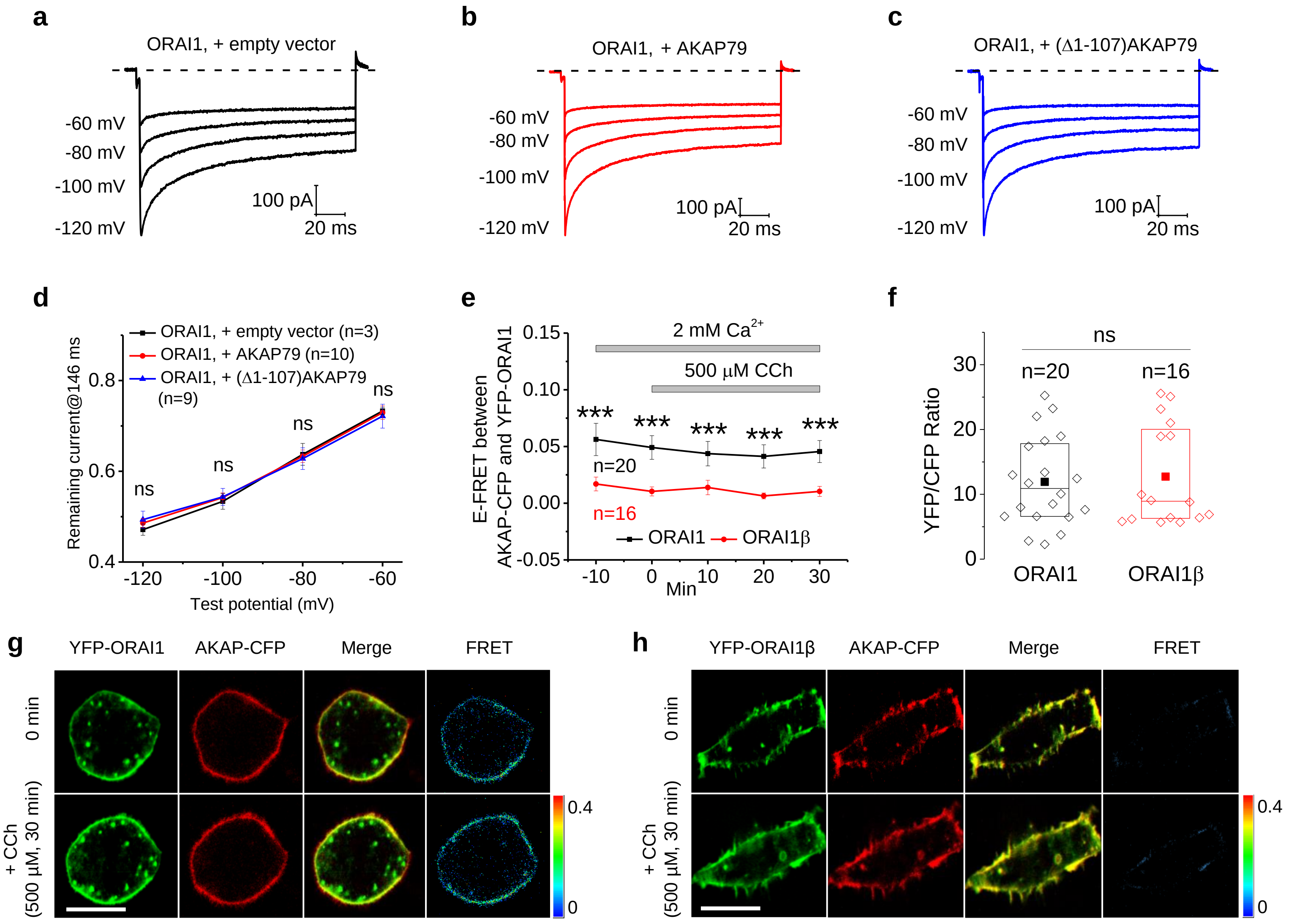

Supplemental Figure 5

Supplementary Figure 5. AKAP79 preferentially interacts with ORAI1.

(a-d) Representative currents from ORAI-KO cells co-expressing eYFP-STIM1 with ORAI1-CFP, and either (a) control cDNA vector, (b) cDNA encoding full length AKAP79, or (c) Δ1-107 AKAP79 cDNA. (d) The extent of CDI is represented as current remaining at 146 ms. Each point represents mean ± SEM for *n* = 3-10 cells. ns, not significant, One-Way ANOVA was used.

(e-h) (e) e-FRET data of interactions between AKAP79-CFP and either eYFP- ORAI1 or eYFP-ORAI1β before and after addition of carbachol (Cch, 500 µM) with each point representing mean ± SEM for *n* = 16-20 cells. ***$p<0.001$, two-tailed Student's t test. (f) YFP/CFP fluorescence ratios from recordings in (e). Boxplots show the mean, median, and the 75th to 25th percentiles of the ratios, ns, not significant; two-tailed Student's t test was used. Representative images of fluorescence and FRET from cells expressing (g) AKAP79-CFP and e-YFP- ORAI1, or (h) AKAP79-CFP and eYFP-ORAI1β. Scale bar: 10µm.

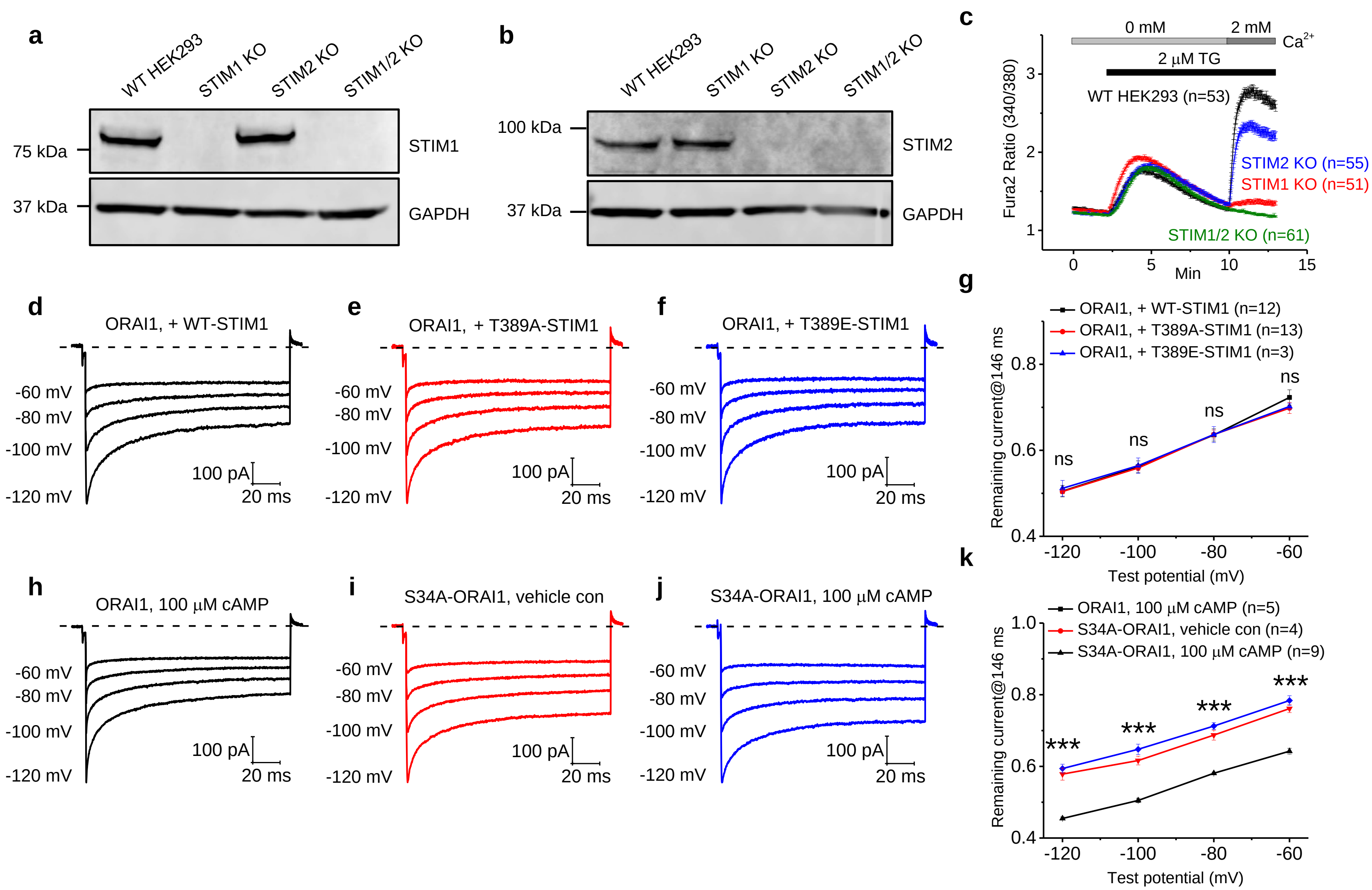

Supplemental Figure 6

Supplementary Figure 6. PKA-mediated phosphorylation of Thereonine-389 of STIM1 is not involved in ORAI1 CDI.

(a-c) (a) STIM1 and (b) STIM2 Western blots on parental WT HEK293 cells, STIM1 KO, STIM2 KO and STIM1/STIM2 double KO cells. (c) Ca^{2+} imaging of SOCE activated by 2 µM thapsigargin (TG) using the dye Fura2 in WT HEK293 cells, STIM1 KO, STIM2 KO and STIM1/STIM2 double KO.

(d-g) Representative currents from STIM1/STIM2 double KO cells co-expressing ORAI1-CFP with either (d) WT STIM1-pVenus, (e) T389A STIM1-pVenus, or (f) T389E STIM1-pVenus. (g) The extent of CDI is represented as current remaining at 146 ms. Each point represents mean ± SEM for n = 3-13 cells. ns, no significant, One-Way ANOVA. Please note the small sample size in the STIM1 T389E condition (n=3). As previously reported[1], only a small portion of cells expressing STIM1 T389E mutant (3 out of 22 cells) yielded large CRAC currents higher than 200 pA (371-478 pA range), allowing us to accurately gauge CDI. The majority of cells (19 out of 22) gave undetectable or small CRAC currents in the 17-126 pA range and were therefore not included in the analysis.

(h-k) Representative currents from ORAI-KO cells co-expressing eYFP-STIM1 with either (h) ORAI1-CFP, or (i, j) S34A ORAI1-CFP. In (h, j) cells were dialyzed with cAMP (100 µM) through the patch pipette while in (i) cells were dialyzed with vehicle control. (k) The extent of CDI is represented as current remaining at 146 ms. Each point represents mean ± SEM for n =4-9 cells. ***p<0.001; One-Way ANOVA was used.

1 Thompson, J. L. & Shuttleworth, T. J. Anchoring protein AKAP79-mediated PKA phosphorylation of STIM1 determines selective activation of the ARC channel, a store-independent Orai channel. *J Physiol* **593**, 559-572, doi:10.1113/jphysiol.2014.284182 (2015).PMC4324705

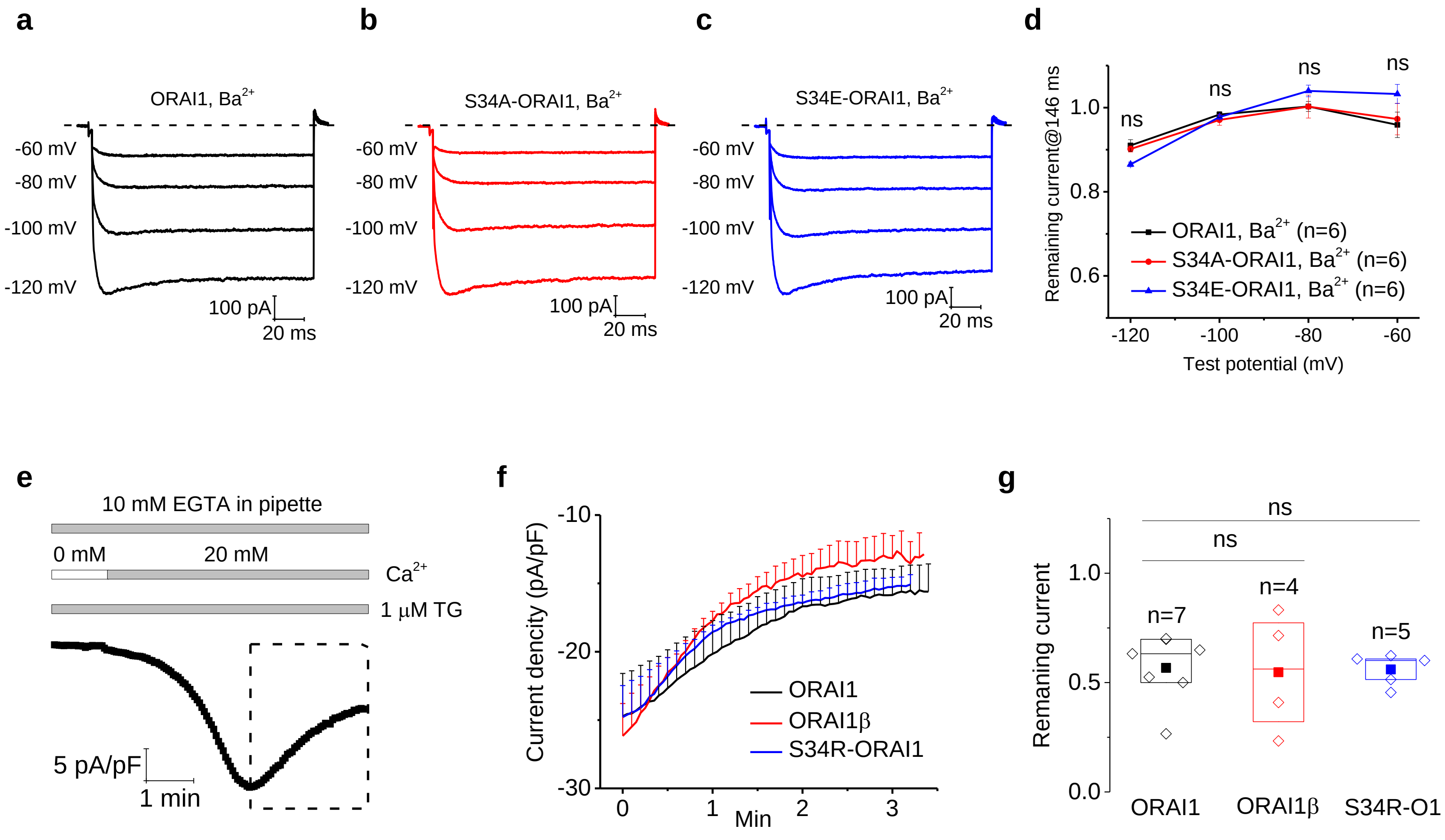

Supplemental Figure 7

Supplementary Figure 7. ORAI1, ORAI1β and S34R-ORAI1 show no differences in slow inactivation driven by global cytosolic Ca^{2+}.

(a-d) Representative currents recorded in Ba^{2+}-containing bath solutions (20 mM) from ORAI-KO cells co-expressing eYFP-STIM1 with either (a) ORAI1-CFP, (b) S34A ORAI1-CFP, or (c) S34E ORAI1-CFP. (d) The extent of CDI is represented as current remaining at 146 ms. Each point represents mean ± SEM for n = 6 cells. ns, not significant; One-Way ANOVA was used.

(e) Slow inactivation driven by global cytosolic Ca^{2+} rise measured from ORAI-KO cells co-expressing eYFP-STIM1 with ORAI1-CFP in the presence of 10mM EGTA in the patch pipette and 20 mM Ca^{2+} and 1 µM thapsigargin (TG) in the bath solution. Dashed box highlights kinetics of this slow inactivation from maximal peak current.

(f, g) (f) Comparison of kinetics of slow inactivation (as in dashed box in (e)) between ORAI1-CFP, ORAI1β-CFP and S34R ORAI1-CFP. (g) Boxplots show the mean, median, and the 75th to 25th percentiles of remaining current 3-min after peak from 4-7 cells per condition. ns, not significant; two-tailed Student's t test was used.

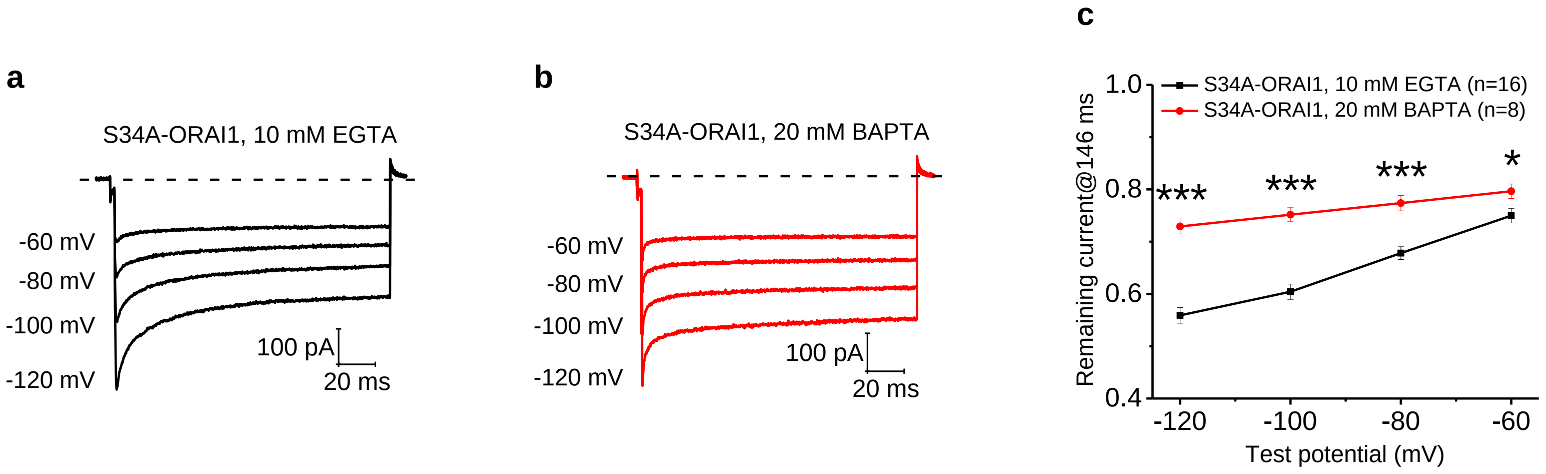

Supplemental Figure 8

Supplementary Figure 8. S34A-ORAI1 show differences in fast CDI when 10 mM EGTA is used in pipette versus 20 mM BAPTA.

(a-c) Representative currents from ORAI-KO cells co-expressing eYFP-STIM1 with S34A-ORAI1-CFP. In (a) cells were dialyzed with 10 mM EGAT through the patch pipette while in (b) cells were dialyzed with 20 mM BAPTA. (c) The extent of CDI is represented as current remaining at 146 ms. Each point represents mean ± SEM for *n* =8-16 cells. *p<0.05; ***p<0.001; two-tailed Student's t test was used.

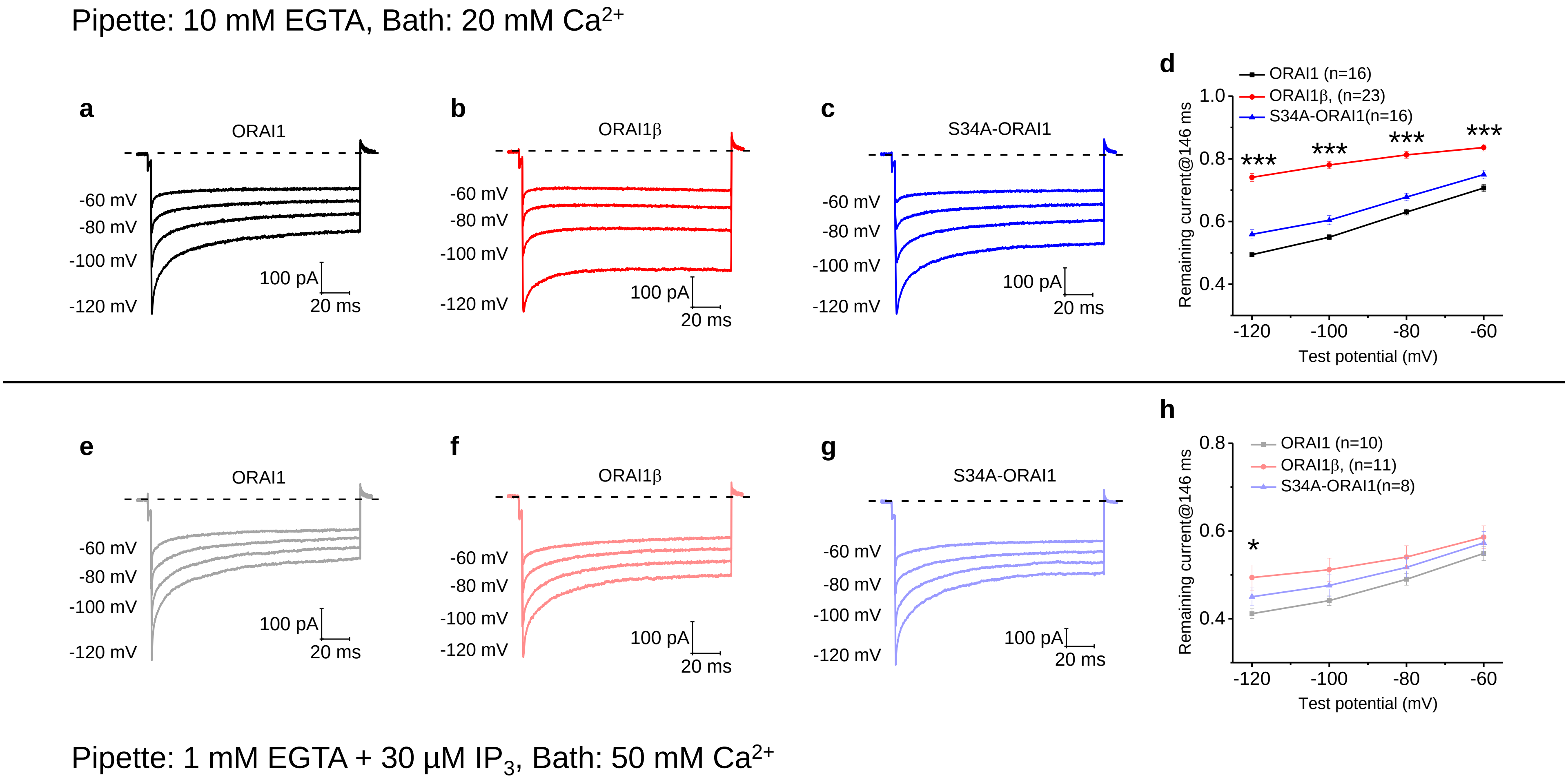

Supplemental Figure 9

Supplementary Figure 9. Differences in CDI between ORAI1, ORAI1β and S34A-ORAI1 persist under conditions of low intracellular buffer and enhanced Ca^{2+} driving force.

(a-d) Representative currents from ORAI-KO cells co-expressing eYFP-STIM1 with either ORAI1-CFP (a), ORAI1β-CFP (b) or S34A-ORAI1-CFP (c) where cells were dialyzed by 10 mM EGTA through the patch pipette and external bath solutions contained 20 mM Ca^{2+}. (d) The extent of CDI is represented as current remaining at 146 ms. Each point represents mean ± SEM for *n* =16-23 cells. ***p<0.001; One-Way ANOVA was used.

(e-h) Representative currents from ORAI-KO cells co-expressing eYFP-STIM1 with either ORAI1-CFP (e), ORAI1β-CFP (f) or S34A-ORAI1-CFP (g) where cells were dialyzed by 1 mM EGTA through the patch pipette (and 30μM 1,4,5-IP_3 to deplete ER stores) and external bath solutions contained 50 mM Ca^{2+}. (h) The extent of CDI is represented as current remaining at 146 ms. Each point represents mean ± SEM for *n* =8-11 cells. *p<0.05; One-Way ANOVA was used.

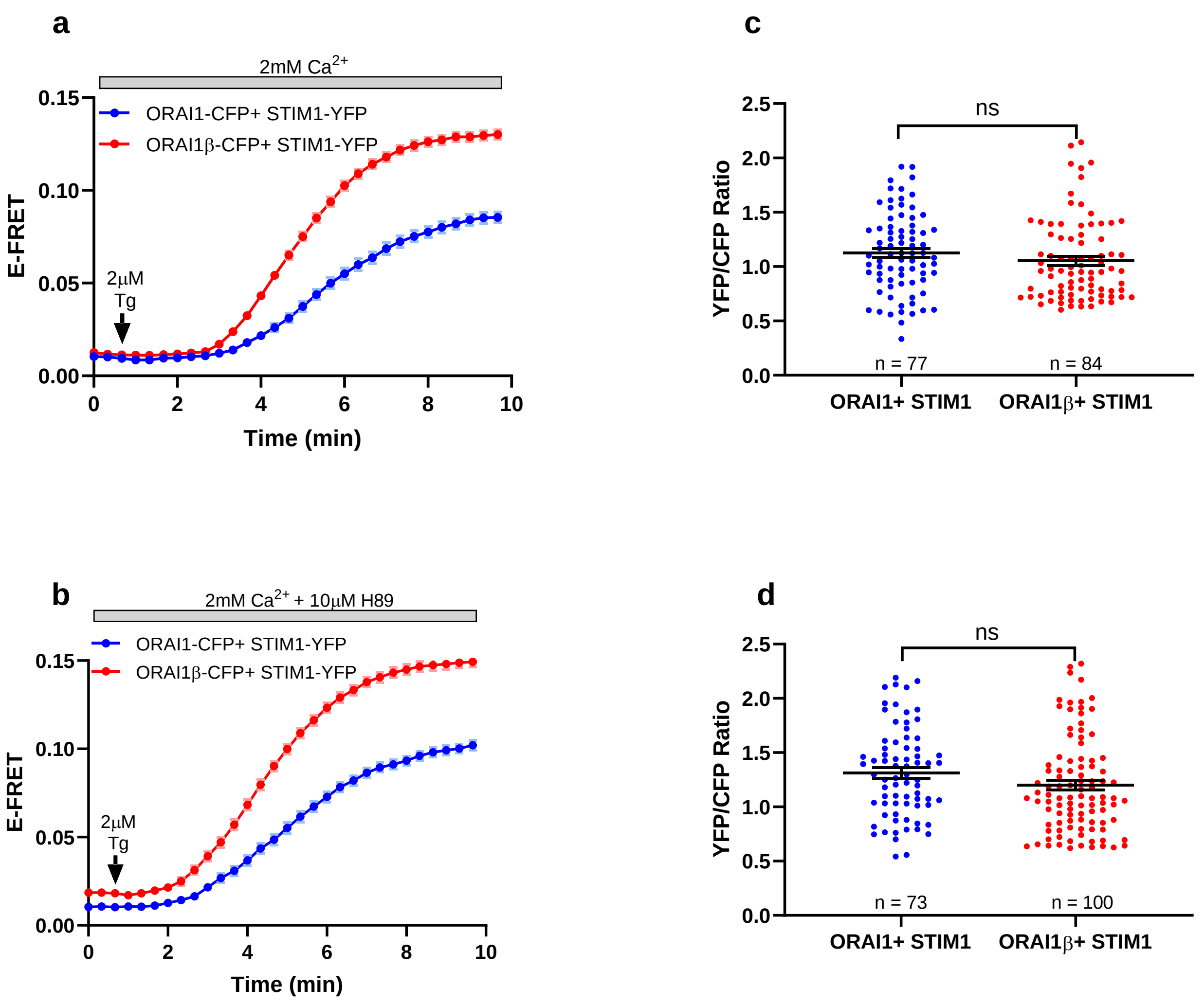

Supplemental Figure 10

Supplementary Figure 10. STIM1/ORAI1 and STIM1/ORAI1β FRET interactions.

(a-d) STIM1/ORAI1 and STIM1/ORAI1β interactions were measured using FRET microscopy under basal conditions and after store depletion with 2µM thapsigargin (a; YFP/CFP ratios shown in c). Similar experiments were performed except that cells were pre-incubated for 5 min with the PKA inhibitor H89 at 10µM (b; YFP/CFP ratios shown in d). Data represent averages from a total of 73-100 cells analyzed from 6 independent runs of each of the four experimental conditions. The time course of E-FRET is represented as mean ± SEM (a, b) from those 73-100 cells and the YFP/CFP ratios represented (c, d) were calculated from the same cells. ns, not significant; two-tailed Student's t test was used.

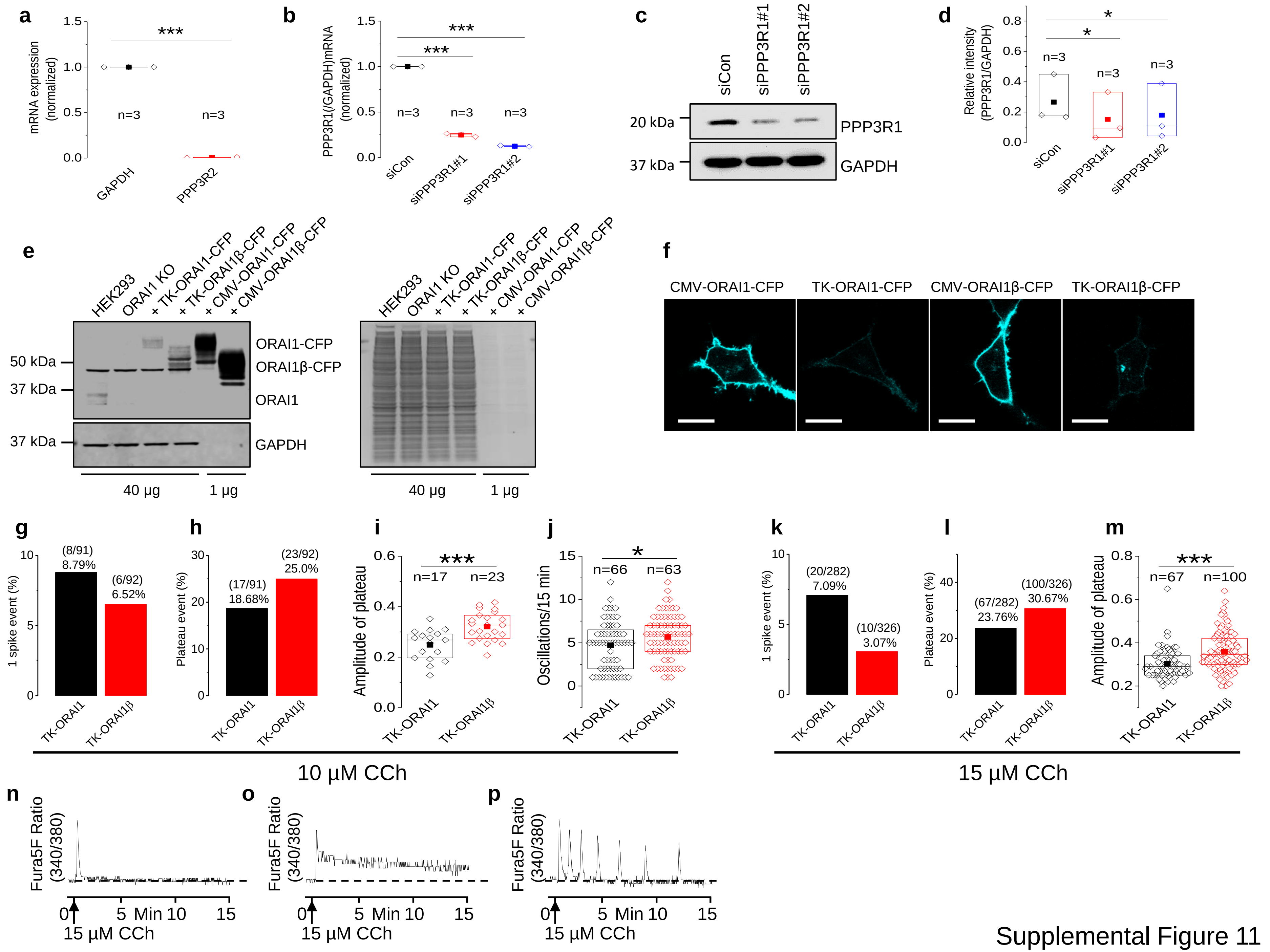

Supplemental Figure 11

Supplementary Figure 11. Under physiological levels of ORAI1 expression and agonist stimulation (10-15 µM Cch), the Ca^{2+} signaling signatures of ORAI1 and ORAI1β are distinct.

(a-d) (a) RT-qPCR showing undetectable levels of PPP3R2 in HEK293 cells. Both siRNA against PPP3R1 used in study significantly reduced PPP3R1 (b) mRNA and (c) protein. (d) Quantification of PPP3R1 protein from 3 independent Western blots as shown in (c). Boxplots show the mean, median, and the 75th to 25th percentile range.

(e, f) Ant-ORAI1 Western blots and corresponding SDS-PAGE gels stained with Coomassie blue showing the differences in protein expression of ORAI1 isoforms when driven by the TK versus the CMV promoter in ORAI1-KO cells. Please note the differences in protein loading (40µg for TK vs 1µg for CMV). (f) Corresponding CFP fluorescence images of ORAI-KO cells transfected with either ORAI1-CFP or ORAI1β-CMV driven by either the TK or the CMV promoter. Scale bar: 10 µm.

(g-j) Summary of cytosolic Ca^{2+} oscillations activated by 10 µM carbachol (Cch) and measured with the dye Fura5F in ORAI1-KO cells expressing either ORAI1-CFP, or ORAI1β-CFP under the control of TK promoter (without STIM1 co-expression). Data represents (g) % cells showing only one initial spike, (h) % cells that respond with a plateau, (i) Boxplots show the mean, median, and the 75th to 25th percentiles of the amplitude of the plateau, and (j) Boxplots show the mean, median, and the 75th to 25th percentiles of oscillations number/15 min from cells showing regenerative Ca^{2+} oscillations. Number of cells for each condition are shown.

(k-m) Data similar to (g-i) from the same cells and under the same conditions but with stimulation with 15 µM carbachol (Cch). Boxplots show the mean, median, and the 75th to 25th percentiles of oscillations number/15 min.

(n-p) Representative traces for 1-spike event (n), plateau (o) and regenerative oscillations (p) are shown.

*$p<0.05$; **$p<0.01$; ***$p<0.001$; two-tailed Student's t test was used for (a, b, d, i, j, m).
