# Supplementary material for: A calcium/cAMP signaling loop at the ORAI1 mouth drives channel inactivation to shape NFAT induction
Source: Nat Commun. 2019 Apr 29;10:1971. doi: 10.1038/s41467-019-09593-0 (PMC6488650; doi:10.1038/s41467-019-09593-0)
Supplement: Supplementary file 2 — Reporting Summary [file 41467_2019_9593_MOESM2_ESM.pdf]

nature research

Corresponding author(s): Mohamed Trebak

Reporting Summary

Nature Research wishes to improve the reproducibility of the work that we publish. This form provides structure for consistency and transparency in reporting. For further information on Nature Research policies, see Authors & Referees and the Editorial Policy Checklist.

Statistical parameters

When statistical analyses are reported, confirm that the following items are present in the relevant location (e.g. figure legend, table legend, main text, or Methods section).

n/a	Confirmed	
☐	☒	The exact sample size (n) for each experimental group/condition, given as a discrete number and unit of measurement
☐	☒	An indication of whether measurements were taken from distinct samples or whether the same sample was measured repeatedly
☐	☒	The statistical test(s) used AND whether they are one- or two-sided<br>*Only common tests should be described solely by name; describe more complex techniques in the Methods section.*
☒	☐	A description of all covariates tested
☒	☐	A description of any assumptions or corrections, such as tests of normality and adjustment for multiple comparisons
☐	☒	A full description of the statistics including central tendency (e.g. means) or other basic estimates (e.g. regression coefficient) AND variation (e.g. standard deviation) or associated estimates of uncertainty (e.g. confidence intervals)
☐	☒	For null hypothesis testing, the test statistic (e.g. F, t, r) with confidence intervals, effect sizes, degrees of freedom and P value noted<br>*Give P values as exact values whenever suitable.*
☒	☐	For Bayesian analysis, information on the choice of priors and Markov chain Monte Carlo settings
☒	☐	For hierarchical and complex designs, identification of the appropriate level for tests and full reporting of outcomes
☒	☐	Estimates of effect sizes (e.g. Cohen's d, Pearson's r), indicating how they were calculated
☐	☒	Clearly defined error bars<br>*State explicitly what error bars represent (e.g. SD, SE, CI)*

Our web collection on statistics for biologists may be useful.

Software and code

Policy information about availability of computer code

Data collection	Clampex 10.3 was used to collect patch clamp data.<br>InCytim2 V5.35 was used to collect Ca2+ imaging data.<br>LAS X Life Science Microscope Software was used to collect some of fluorescent imaging data.<br>SlideBook 6.0 was used to collect FRET data.<br>MAXCHELATOR website was used to calculate free Ca2+ concentration for pipette solution.
Data analysis	Origin 9.0 was used to analyze patch clamp data and Ca2+ imaging data.<br>Clampfit 10.3 was used to analyze patch clamp data.<br>ImageJ was used to analyze western blot data.

For manuscripts utilizing custom algorithms or software that are central to the research but not yet described in published literature, software must be made available to editors/reviewers upon request. We strongly encourage code deposition in a community repository (e.g. GitHub). See the Nature Research guidelines for submitting code & software for further information.

Data

Policy information about availability of data

All manuscripts must include a data availability statement. This statement should provide the following information, where applicable:

- Accession codes, unique identifiers, or web links for publicly available datasets
- A list of figures that have associated raw data
- A description of any restrictions on data availability

Source information related to KEY RESOURCES AND REAGANTS, including antibodies, chemicals, peptides commercial assays, cell lines, oligonucleotides, recombinant DNAs, software and algorithms are provided in Supplementary Table 1. Source data for real time-PCR primers are provided in Supplementary Table 2. Source data for siRNA sequences are provided in Supplementary Table 3. Source data for patch clamp data are provided in Supplementary Table 4. All other data supporting the findings of this study are available from the corresponding author on reasonable request.

Field-specific reporting

Please select the best fit for your research. If you are not sure, read the appropriate sections before making your selection.

☒ Life sciences ☐ Behavioural & social sciences ☐ Ecological, evolutionary & environmental sciences

For a reference copy of the document with all sections, see nature.com/authors/policies/ReportingSummary-flat.pdf

Life sciences study design

All studies must disclose on these points even when the disclosure is negative.

Sample size	The samples sizes were chosen based on previous studies with similar methodologies.
Data exclusions	Patch clamp data, current size smaller than 200 pA were excluded from the analyses.
Replication	Every single experiments were carried out under clearly defined and standard conditions and were repeated at least twice when ever possible. All replication attempts were successful.
Randomization	n/a
Blinding	n/a

Reporting for specific materials, systems and methods

Materials & experimental systems

n/a	Involved in the study
☐	☒ Unique biological materials
☐	☒ Antibodies
☐	☒ Eukaryotic cell lines
☒	☐ Palaeontology
☒	☐ Animals and other organisms
☒	☐ Human research participants

Methods

n/a	Involved in the study
☒	☐ ChIP-seq
☒	☐ Flow cytometry
☒	☐ MRI-based neuroimaging

Unique biological materials

Policy information about availability of materials

Obtaining unique materials	ORAI1 knockout, STIM1 knockout, STIM2 knockout and STIM1/2 double knockout HEK293 cell lines were generated by ourself using the CRISPR-Cas9 system and are readily available under request upon publication of the study.

Antibodies

Antibodies used	Rabbit polyclonal anti-ORAI1 (Sigma-Aldrich, now MilliporeSigma, Cat#O8264, 1:4000)<br>Mouse monoclonal anti-GAPDH (MilliporeSigma Cat#MAB374, 1:2000)

Rabbit polyclonal anti-ADCY8 (Labome Cat#55065-1-AP, 1:4000)
Mouse monoclonal anti-GFP (R and D Systems-MAB42401, Immunoprecipitation, 2 µg/100 µg cell lysate)
Rabbit polyclonal anti-Phosphoserine (MilliporeSigma Cat#AB1603, 1:1000)
Mouse monoclonal anti-PPP3R1 (Santa Cruz Biotechnology Cat#sc-130393, 1:2000)
Mouse monoclonal anti-HSC70 (Santa Cruz Biotechnology Cat#sc-24, 1:1000)

Validation

anti-ORAI1 (https://www.sigmaaldrich.com/catalog/product/sigma/o8264?lang=en&region=US)
anti-GAPDH (http://www.emdmillipore.com/US/en/product/Anti-Glyceraldehyde-3-Phosphate-Dehydrogenase-Antibody-clone-6C5,MM_NF-MAB374)
anti-ADCY8 (https://www.labome.com/product/Proteintech-Group/55065-1-AP.html)
anti-GFP (https://www.rndsystems.com/products/gfp-antibody-454505_mab42401)
anti-Phosphoserine (http://www.emdmillipore.com/US/en/product/Anti-Phosphoserine-Antibody,MM_NF-AB1603)
anti-PPP3R1 (https://www.scbt.com/scbt/product/pp2b-b1-antibody-70-a?requestFrom=search)
anti-HSC70 (https://www.scbt.com/scbt/product/hsp-70-hsc-70-antibody-w27?requestFrom=search)

Eukaryotic cell lines

Policy information about cell lines

Cell line source(s)

HEK293 cell line was purchased from ATCC. All other knockout cell lines used in this study were generated in our lab using the CRISPR-Cas9 system

Authentication

Authentication was not performed as none of the cell lines used in this study have been listed in the commonly misidentified lines.

Mycoplasma contamination

All cell lines were regularly tested for mycoplasma using the abm PCR Mycoplasma Detection Kit (abm), Catalog #: G238.

Commonly misidentified lines
(See ICLAC register)

No misidentified lines were used in this study.
